# Dietary and other lifestyle correlates of serum folate concentrations in a healthy adult population in Crete, Greece: a cross-sectional study

**DOI:** 10.1186/1475-2891-5-5

**Published:** 2006-02-10

**Authors:** Christos M Hatzis, George K Bertsias, Manolis Linardakis, John M Scott, Anthony G Kafatos

**Affiliations:** 1 Preventive Medicine and Nutrition Clinic, Department of Social Medicine, Faculty of Medicine, University of Crete, P.O. Box 1352, 71110, Heraklion, Greece; 2 Biochemistry Department, Trinity College Dublin, Dublin 2, Ireland

## Abstract

**Background:**

Folate has emerged as a key nutrient for optimising health. Impaired folate status has been identified as a risk factor for cardiovascular disease, various types of cancers, and neurocognitive disorders. The study aimed at examining the distribution and determinants of serum folate concentrations in a healthy adult population in Crete, Greece.

**Methods:**

A cross-sectional sample of 486 healthy adults (250 men, 236 women) aged 39 ± 14 years, personnel of the Medical School and the University Hospital of Crete in Greece, was examined. Serum folate and vitamin B_12 _concentrations were measured by microbiological assay, and total homocysteine was determined fluorometrically and by high-pressure liquid chromatography. Lifestyle questionnaires were completed, and nutrient intakes and food consumption were assessed by 24-h dietary recalls. Multivariate analyses were performed using SPSS v10.1.

**Results:**

The geometric mean (95% confidence interval) concentrations of serum folate were 15.6 μmol/l (14.6–16.8) in men and 19.2 μmol/l (17.9–20.7) in women (p < 0.001). Inadequate folate levels (≤7 nmol/l) were present in 6.8% of men and 2.1% of women (p < 0.001). Approximately 76% of men and 87% of women did not meet the reference dietary intake for folate (400 μg/day). Serum folate was inversely related to total homocysteine levels (p < 0.001). Increased tobacco and coffee consumption were associated with lower folate concentrations (p < 0.05 for both) but these associations disappeared after controlling for nutrient intakes. In multivariate analysis, intakes of MUFA, fibre, calcium, magnesium, folate, and vitamins A, E, C, B_1_, and B_6 _were positively associated with serum folate. Consumption of potatoes, legumes, fruits, and vegetables were favourably related to the serum folate status.

**Conclusion:**

Serum folate concentrations were associated with various demographic, lifestyle and dietary factors in healthy Cretan adults. Large-scale epidemiological studies should be conducted within the general Greek adult population to assess the prevalence of impaired folate status and further examine associations with dietary patterns and chronic disease risk. Considering the importance of folate in health maintenance, it is important to increase the public's awareness of modifiable lifestyle patterns and diet and tobacco use in particular, which may be associated with improved folate status.

## Background

Dietary habits in a population may be a determining factor of health, and inadequate intakes of certain nutrients have been associated with increased risk for chronic disease, such as cardiovascular disease, diabetes, and cancer. More recently, folate has drawn attention as a key nutrient involved in health maintenance and chronic disease prevention [1]. Folate acts as a coenzyme in several single-carbon transfers leading to the biosynthesis of nucleic acids, certain neurotrasmitters, phospholipids, and hormones. It is involved in homocysteine metabolism, donating a methyl group during homocysteine remethylation into methionine, and inadequate folate status has been characterized as a major cause of hyperhomocysteinaemia [2]. Moreover, folate deficiency is an established risk factor for the development of certain types of cancer, cardiovascular disease [3,4], developmental defects (e.g. neural tube defects), and neurological or psychiatric disorders [5-7]. Although much of the adverse effects of impaired folate status is explained through its interplay with homocysteine levels, there is evidence suggesting direct health-beneficial effects of folate, including its favourable effects on vascular endothelial function and redox cellular status [1].

From a public health perspective, assessing the folate status in a population and examining its dietary or other correlates, is of great importance. This would allow the identification of groups at risk for folate deficiency that would mostly benefit from public health interventions to increase dietary folate intake.

Crete (Greece), once known for the low cardiovascular mortality among adult men in early 1950's, has now increasing rates of cardiovascular disease, a trend that appears to be related to dietary and lifestyle changes that have been taking place during the last decades [8,9]. The traditional Cretan diet – a variant of the Mediterranean diet – has been gradually abandoned, and current Cretans consume higher amounts of saturated fat, meat, and cheese, and lower amounts of bread, fruits, vegetables, legumes, and fibre [9-11]. Among other consequences, such dietary changes are expected to result in decreased intake of dietary folate and thus, impaired folate status. To date, however, no data are available with regard to the folate status of the adult population in Crete.

In this study, we report on the folate status of a group of apparently healthy Cretan adults, personnel of the University of Crete and the University Hospital of Heraklion (Crete), which were examined as part of a health prevention programme by the Preventive Medicine and Nutrition Clinic. We describe the distribution of serum folate concentrations by age and sex, and investigate associations with nutrient intakes, food consumption, and lifestyle habits, namely tobacco, coffee, and alcohol consumption. Our findings document certain patterns of dietary intake and lifestyle habits associated with folate levels, with potential implications for the development of future health intervention studies.

## Methods

### Study sample

The study was conducted as part of a health prevention programme undertaken by the Preventive Medicine and Nutrition Clinic at the University of Crete School of Medicine. The personnel of University Hospital of Heraklion (Crete, Greece) and the University of Crete School of Medicine were asked to participate in the health assessment survey by written announcements. Subjects were excluded if they had history of any chronic disease such as cancer, diabetes mellitus, hypertension, liver or coronary heart disease. The study sample consisted of 381 (out of 2445) employees (184 men, 197 women, aged 20–72 years) and 105 (out of 155) (66 men, 39 women, aged 20–40 years) practicing medical students.

### Laboratory measurements

Early morning venous blood samples were drawn for biochemical screening tests, following a 12-hour overnight fast. The blood samples (10 ml) were transferred to the Nutritional Research Laboratory of the University of Crete in tanks containing ice packs so as to maintain a temperature of 3–4°C. Blood was centrifuged and 1.5 ml aliquots were pipetted into plastic Eppendorf tubes. One aliquot was used for blood analysis of triacylglycerol (TG), total cholesterol (TC) and high-density lipoprotein cholesterol (HDL-C) measurements [12] on the same day of collection, while the other was stored (at -80°C) for determination of serum folate, vitamin B_12_, and total homocysteine. Serum samples were sent in dry ice to Trinity College of Dublin (Ireland), and serum folate and vitamin B_12 _concentrations were measured by a 96-well plate microbiological assay [13,14]. Serum total homocysteine concentrations were determined fluorometrically and by high-pressure liquid chromatography (HPLC) [15].

### Health assessment questionnaires

Purpose designed questionnaires were administered to ascertain biographical data, lifestyle behaviours on topics including cigarette smoking and medical history [12]. To validate the results, multiple crosschecked questions on the same topic were addressed to the participants. Smokers were classified as those who stated smoking more than one cigarette per day for at least three consecutive months. Ex-smokers were defined as those who had not been smoking for the last six consecutive months, and non-smokers as those who did not fall in any of the two previous groups.

### Dietary survey

Dieticians administered a 24 h dietary recall to all participants. Detailed descriptions of all foods, beverages and supplements consumed during the 24 h period before the interview, including the quantity, cooking method and brand names were recorded. Dietary records were collected from 383 participants (197 men, 186 women). Food quantities were assessed by the use of household measures and colour food-model photographs. The 24 h dietary recall has been previously validated in relation to fat intake based on measurements with adipose tissue aspiration and fatty acid composition analysis [8]. Nutrient contents were analysed according to the food database developed at the Department of Social Medicine of the University of Crete in 1998 and updated in 2000 [11]. The database includes about 500 foods, both single and composite. The macro- and micro-nutrient composition of about 20 foods has been chemically determined at Wageningen Agricultural University. The fatty acid content of 105 fat-containing foods was determined at the TNO Nutrition and Food Research Institute (The Netherlands) during 1997. For the remainder of fat-containing foods, the fatty acid analyses were drawn from the analyses available within the European "trans fatty acid research project" database (TTDB, version 1.2) developed at the TNO Nutrition and Food Research Institute between 1995 and 1997. For the foods whose composition was not chemically determined, values from the US Department of Agriculture database v11.1 were used. Recipe calculations were used for composite Greek foods with the ingredients being weighed prior to and also following cooking in each case.

### Statistical analyses

The distribution of serum folate was markedly skewed toward high values and was corrected by log transformation. The geometric means and their 95% confidence intervals (CI) are presented. Variables not normally distributed were also log-transformed. We tested for statistically significant differences between men and women using one-way analysis of variance (ANOVA). The chi-squared (*χ*^2^) test was used for categorical variables. Serum folate concentrations were considered sub-optimal when ≤7 nmol/l [16]. To estimate mean total homocysteine and vitamin B_12 _concentrations across levels of serum folate, age and sex-specific quartiles were calculated. Geometric mean (95% CI) folate concentrations within categories of tobacco, coffee, and alcohol consumption were determined by analysis of covariance (ANCOVA), controlling for age, gender, district of residence (rural/urban), energy intake, and intakes of several nutrients which were significantly associated to serum folate (MUFA, fibre, calcium, magnesium, folate, vitamins A, E, C, B_1_, B_6_). Linear contrasts were applied to test for trends across groups with ≥3 groups and collinearity was assessed by calculation of variance inflation factors (VIF).

Nutrient intakes were calculated across age and sex-specific quartiles of serum folate, and Pearson's partial correlation coefficients (*r*) were used to evaluate linear associations between nutrient intakes and serum folate after controlling for age, gender, district of residence, body mass index, energy intake, and consumption of tobacco, coffee, and alcohol. Finally, individuals were categorized according to levels of consumption of various foods (none, below or above median consumption) and levels of serum folate (below or above 1^st ^quartile, age- and gender-specific). Logistic regression was used to calculate odds ratio (95% CI) for low serum folate (<1^st ^quartile) according to consumption of foods, including district of residence, body mass index, energy intake, and consumption of tobacco, coffee, and alcohol as independent variables. All the *p *values reported are two-tailed and statistical significance was defined as *p *< 0.05. Statistical analyses were performed using the Statistical Package for Social Sciences (SPSS for Windows 11.5.0, SPSS, Inc.).

## Results

The mean ± standard deviation (SD) age was 39 ± 14 years for men and 39 ± 13 years for women (Table 1). Approximately 86% of the subjects lived in urban areas and current smokers were the 38% of men and 31% of women (*p *> 0.05).

**Table 1:** Demographics and other characteristics of the study sample of Cretan adults.

	Men (N = 250)	Women (N = 236)	
	Mean (SD) or %		*p *value^1^

Age (years)	39 (14)	39 (13)	-^2^
District of residence (% urban)	86.4	85.6	-
Tobacco use (% current use)	37.7	31.0	-
BMI (kg/m^2^)	26.8 (3.5)	26.1 (5.8)	<0.001
Systolic blood pressure (mmHg)	129.4 (16.7)	124.2 (20.3)	<0.001
Diastolic blood pressure (mmHg)	84.3 (10.0)	79.2 (10.9)	<0.001
Total cholesterol (mg/dL)	224.0 (50.3)	221.7 (54.0)	-
Triglycerides (mg/dL)	128.2 (83.3)	99.9 (56.9)	<0.001
HDL-cholesterol (mg/dL)	45.3 (9.8)	56.5 (12.2)	<0.001
Energy intake (kcal/day) ^3^	2133 (793)	1605 (629)	<0.001
Total fat (% energy)^3^	39.7 (9.8)	40.2 (11.1)	-
SFA (% energy) ^3^	11.5 (5.0)	11.1 (4.9)	-
Dietary cholesterol (mg/day)^3^	213.7 (157.4)	153.6 (130.9)	<0.001
Dietary fibre (g/day)^3^	22.1 (11.3)	18.7 (11.0)	0.001

^1 ^Analysis of variance (ANOVA) or chi-squared test.

^2 ^Not statistically significant.

^3 ^N = 197 for men and N = 186 for women

The distribution of serum folate concentrations and dietary folate intakes is presented for all subjects by gender and age in Table 2. The geometric mean of serum folate was 15.6 nmol/l (95% CI, 14.6–16.8 nmol/l) in men and 19.2 nmol (17.9–20.7 nmol/l) in women (*p *< 0.001). In both men and women age was positively associated with serum folate. The prevalence of sub-optimal serum folate (≤7 nmol/l) was 6.8% in men and 2.1% in women (*p *< 0.001). Men had higher mean intake of dietary folate than women (294 vs. 247 μg/d, *p *= 0.003). However, the energy-adjusted intake of folate was higher in women than men (161 vs. 144 μg/1000 kcal/d, *p *> 0.05). Eighty-seven percent of women and 76% of men (*p *= 0.013) consumed folate <400 μg/day. Dietary intake of folate increased by age, especially in men (*p *for trend < 0.01).

**Table 2:** Serum folate concentrations and dietary intake of folate in the study sample of Cretan adults.^1^

		Serum folate	% ≤7		Folate intake		% <400
		(nmol/l)^2^	nmol/l		(μg/d)^3^	(μg/1000 kcal/d)	μg/d

Men							
20–34 y	(n = 107)	13.8 (12.5–15.3)	7.5	(n = 103)	248 (213–283)	119 (106–133)	83.5
35–50 y	(n = 80)	16.0 (14.2–18.0)	6.3	(n = 72)	351 (297–406)	174 (149–198)	69.4
>50 y	(n = 63)	18.6 (15.8–21.9)^4^	6.3	(n = 20)	325 (242–407)^5^	166 (130–201)^6^	65.0
Total	(n = 250)	15.6 (14.6–16.8)	6.8	(n = 195)	294 (265–323)	144 (132–157)	76.4
							
Women							
20–34 y	(n = 101)	17.4 (15.8–19.2)	3.0	(n = 96)	247 (207–288)	159 (134–184)	86.5
35–50 y	(n = 79)	19.4 (17.2–22.0)	1.3	(n = 72)	242 (198–287)	155 (133–177)	88.9
>50 y	(n = 56)	22.8 (19.3–26.9)^7^	1.8	(n = 17)	266 (160–372)	192 (129–255)	76.5
Total	(n = 236)	19.2 (17.9–20.7)	2.1	(n = 185)	247 (219–275)	161 (144–177)	86.5

^1 ^Data are presented as geometric mean (95% confidence interval).

^2 ^Men had significantly lower serum folate concentrations than women (*p *< 0.001).

^3 ^Men had significantly higher intake of dietary folate (μg/d) than women (*p *= 0.003).

^4 ^*p *= 0.001 (trend by age).

^5 ^*p *= 0.003 (trend by age).

^6^*p *< 0.001 (trend by age).

^7^*p *= 0.008 (trend by age).

Associations between age and sex-specific quartiles of serum folate and mean levels of total homocysteine and vitamin B_12 _are presented in Table 3. Serum folate showed an inverse linear association with total homocysteine (*p *for trend < 0.001), and subjects at the highest folate quartile had 18.5% lower mean total homocysteine concentrations compared to those at the lowest quartile. No significant association was observed between serum folate and vitamin B_12 _levels.

**Table 3:** Association between serum total homocysteine and vitamin B_12 _concentrations and quartiles (age and sex-specific) of serum folate in Cretan adults.

	Serum folate quartiles (age & gender-specific)				
	Q1	Q2	Q3	Q4	

	Geometric mean				*p *for trend^1^

Serum total homocysteine (μmol/L)	12.4	10.3	10.2	10.1	<0.001
Serum vitamin B_12 _(pmol/L)	307.5	305.2	310.4	286.1	-^2^

^1 ^ANOVA (linear contrasts).

^2 ^Not statistically significant.

The relations between serum folate and the use of tobacco, coffee, and alcohol are shown in Table 4. The age- and gender-adjusted geometric mean serum folate was ≈2 nmol/l lower in current smokers than in non- or ex-smokers (*p *< 0.05). Similarly, serum folate levels were inversely related to the quantity of tobacco (*p *for trend < 0.05), and individuals who smoked ≥20 cigs/day had 21.2% lower mean folate concentrations compared to those who smoked <10 cigs/day. Increasing coffee intake was also related to decreased serum folate (*p *for trend < 0.05), and subjects consuming >200 g/day had 14.8% lower mean serum folate levels compared to non-consumers. However, the associations between tobacco or coffee consumption and serum folate disappeared after adjusting for intakes of nutrients, especially dietary folate, vitamins A and C (multivariate-adjusted). No significant relationship was observed between alcohol intake and serum folate.

**Table 4:** Association between serum folate concentrations and consumption of tobacco, coffee, and alcohol in the study sample of Cretan adults.

	Age- & gender-adjusted ^1^	Multivariate-adjusted ^2^
	Geometric mean (95% CI)	

Tobacco consumption		
Non/ex-smokers (n = 314)	18.0 (16.9–19.1)	17.4 (16.3–18.6)
Current smokers (n = 165)	16.0 (14.7–17.4)^3^	16.2 (14.9–17.7)
<10 cigs/day (n = 52)	17.7 (15.3–20.5)	16.5 (14.1–19.4)
10–19 cigs/day (n = 36)	15.0 (12.6–17.9)	14.7 (12.2–17.8)
≥20 cigs/day (n = 76)	14.6 (12.9–16.5)^4^	14.9 (12.9–17.2)
		
Coffee consumption		
None (n = 102)	18.2 (16.4–20.2)	17.9 (16.2–19.8)
≤200 g/day (n = 195)	16.9 (15.7–18.2)	17.2 (16.0–18.5)
>200 g/day (n = 86)	15.5 (13.8–17.3)^5^	15.6 (14.0–17.4)
		
Alcohol consumption		
None (n = 293)	17.0 (16.0–18.1)	17.0 (16.0–18.0)
≤180 g/day (n = 43)	16.0 (13.6–18.9)	16.1 (13.8–18.9)
>180 g/day (n = 47)	16.8 (14.4–19.7)	18.2 (15.6–21.2)

^1 ^ANCOVA, controlling for age and gender.

^2 ^ANCOVA, controlling for age, gender, district of residence, total energy intake, intakes of MUFA, fibre, calcium, magnesium, folate, vitamins A, E, C, B_1_, B_6 _(all log transformed).

^4 ^Non/ex-smokers had significantly higher serum folate concentrations than current smokers (*p *= 0.029).

^5 ^*p *= 0.047, linear trend by level of tobacco consumption.

^6 ^*p *= 0.035, linear trend by level of coffee consumption.

The associations between nutrient intakes and serum folate are shown in Table 5. In multivariate analysis, intake of mono-unsaturated fatty acids (MUFA) – both as raw quantity and as percentage of energy intake – was positively associated with serum folate. Intakes of dietary fibre (*r *= 0.20), calcium (*r *= 0.14), and magnesium (*r *= 0.10) were also positively related to serum folate, independently of age, gender, energy intake, consumption of tobacco, coffee, and alcohol. Serum folate concentrations demonstrated favourable associations with dietary intakes of several vitamins, namely folate (*r *= 0.14), vitamins A (*r *= 0.23), E (*r *= 0.15), C (*r *= 0.21), B_1 _(*r *= 0.13), and B_6 _(*r *= 0.12).

**Table 5:** Mean nutrient intakes in relation to serum folate quartiles in the study sample of Cretan adults.

	Serum folate quartiles (age- & gender-specific)				Correlation coefficient (r)	
	Q1	Q2	Q3	Q4	Age/gender-adjusted^1^	Multivariate-adjusted^2^

Energy (kcal)	1700	1769	1595	1647	-0.03	-
Carbohydrates (g)	179.1	184.7	192.7	185.2	-0.01	0.03
% energy	44.5	45.3	47.7	45.4	0.03	0.03
Protein (g)	67.8	60.0	62.4	61.3	-0.06	-0.06
% energy	14.5	13.3	13.2	14.0	-0.06	-0.06
Total fat (g)	72.6	72.5	70.8	72.3	-0.01	0.02
% energy	41.1	40.9	39.5	40.2	0.00	0.00
SFA (g)	19.3	19.6	19.4	18.3	-0.05	-0.05
% energy	11.7	11.6	11.7	11.0	-0.04	-0.05
PUFA (g)	8.8	9.2	8.8	8.7	0.00	0.03
% energy	5.4	5.5	5.3	5.2	0.01	0.01
MUFA (g)	30.1	33.0	31.6	33.7	0.09	0.12^3^
% energy	18.4	19.6	18.9	20.6	0.13 ^3^	0.12^3^
Trans FA (g)	1.4	1.3	1.5	1.1	-0.08	-0.07
n-6 FA (g)	8.8	8.0	8.3	7.4	-0.05	-0.02
n-3 FA (g)	0.9	0.7	0.8	0.6	-0.05	-0.03
Cholesterol (mg)	192.4	170.2	206.1	166.3	-0.04	-0.03
Fibre (g)	13.5	16.3	18.1	18.0	0.17^4^	0.20^5^
Calcium (mg)	549.5	622.9	648.3	697.1	0.10^3^	0.14^3^
Iron (mg)	10.6	10.5	11.6	11.2	0.04	0.08
Magnesium (mg)	233.7	243.5	238.8	258.7	0.04	0.10^3^
Phosphorous (mg)	928.6	946.2	917.2	946.0	-0.03	-0.02
Sodium (mg)	1507	1583	1678	1492	-0.02	0.01
Potassium (mg)	2289	2191	2185	2565	0.08	0.09
Folate (μg)	187.8	211.1	220.9	232.5	0.11^3^	0.14^4^
Niacin (mg)	13.3	13.1	14.0	12.7	-0.03	0.01
Vitamin A (μg)	538.1	546.6	768.3	1011	0.23^5^	0.23^5^
Vitamin E (mg)	6.1	7.0	7.0	7.6	0.15^4^	0.15^4^
Vitamin C (mg)	75.5	80.9	93.4	133.4	0.22^5^	0.21^5^
Vitamin B_1 _(mg)	1.2	1.3	1.4	1.4	0.09	0.13^3^
Vitamin B_2 _(mg)	1.2	1.3	1.4	1.4	0.04	0.06
Vitamin B_6 _(mg)	1.3	1.3	1.3	1.5	0.09	0.12^3^
Vitamin B_12 _(μg)	4.0	3.1	4.0	3.0	-0.03	-0.03

^1 ^Partial correlation coefficient between intakes of nutrients and serum folate concentrations (all log transformed) controlling for age and gender.

^2 ^Partial correlation coefficient between intakes of nutrients and serum folate concentrations (all log transformed) controlling for age, gender, district of residence, body mass index, energy intake, consumption of tobacco, coffee, and alcohol.

^3 ^*p *< 0.05

^4 ^*p *< 0.01

^5 ^*p *< 0.001

We further assessed food consumption by the study participants according to their serum folate status (Table 6). Multivariate logistic regression analysis showed that increased consumption of potatoes, legumes, fruits and/or vegetables were associated with decreased risk for low serum folate (determined as folate concentrations <1^st ^quartile, age- and gender-specific). Specifically, individuals consuming fruits and/or vegetables ≥360 g/day had 79% lower risk for low serum folate, compared to those with no consumption. In contrast, increased intake of cereals and meat (especially red meat) was inversely related to serum folate status.

**Table 6:** Association between foods consumption and serum folate status in the study sample of Cretan adults.

	Odds ratio (95% CI) for low serum folate (<1^st ^quartile, age- & gender-specific)	
	Unadjusted^1^	Multivariate-adjusted^2^

Bread		
None	1.00 (reference)	1.00 (reference)
<90 g	0.81 (0.38–1.73)	0.76 (0.35–1.64)
≥90 g	0.74 (0.35–1.58)	0.71 (0.33–1.52)
	*p *= 0.451	*p *= 0.428
Cereals		
None	1.00 (reference)	1.00 (reference)
<90 g	1.47 (0.82–2.63)	1.46 (0.79–2.68)
≥90 g	1.74 (1.01–3.02)	1.75 (0.98–3.12)
	*p *= 0.045	*p *= 0.057
Potatoes		
None	1.00 (reference)	1.00 (reference)
<122 g	0.54 (0.26–1.12)	0.53 (0.25–1.12)
≥122 g	0.41 (0.19–0.88)	0.42 (0.19–0.91)
	*p *= 0.008	*p *= 0.012
Legumes		
None	1.00 (reference)	1.00 (reference)
<300 g	0.71 (0.28–1.80)	0.73 (0.28–1.88)
≥300 g	0.44 (0.20–1.02)	0.39 (0.16–0.97)
	*p *= 0.042	*p *= 0.037
Vegetables		
None	1.00 (reference)	1.00 (reference)
<190 g	0.71 (0.37–1.35)	0.69 (0.36–1.35)
≥190 g	0.41 (0.21–0.81)	0.39 (0.19–0.78)
	*p *= 0.006	*p *= 0.005
Fruits		
None	1.00 (reference)	1.00 (reference)
<261 g	0.70 (0.40–1.23)	0.67 (0.37–1.21)
≥261 g	0.81 (0.46–1.41)	0.89 (0.50–1.59)
	*p *= 0.425	*p *= 0.645
Fruits and/or vegetables		
None	1.00 (reference)	1.00 (reference)
<360 g	0.42 (0.16–1.08)	0.31 (0.11–0.85)
≥360 g	0.27 (0.10–0.73)	0.21 (0.07–0.60)
	*p *< 0.001	*p *= 0.009
Meat		
None	1.00 (reference)	1.00 (reference)
<100 g	1.30 (0.73–2.33)	1.46 (0.80–2.65)
≥100 g	2.52 (1.43–4.44)	2.51 (1.35–4.65)
	*p *= 0.002	*p *= 0.004
Red meat		
None	1.00 (reference)	1.00 (reference)
<90 g	1.40 (0.74–2.65)	1.49 (0.77–2.90)
≥90 g	2.31 (1.32–4.05)	2.31 (1.28–4.19)
	*p *< 0.001	*p *= 0.005
Eggs		
None	1.00 (reference)	1.00 (reference)
<50 g	1.09 (0.47–2.56)	1.16 (0.49–2.78)
≥50 g	0.45 (0.17–1.19)	0.50 (0.19–1.33)
	*p *= 0.153	*p *= 0.248
Dairy products		
None	1.00 (reference)	1.00 (reference)
<130 g	1.62 (0.86–3.04)	1.50 (0.78–2.90)
≥130 g	1.16 (0.61–2.21)	1.16 (0.60–2.26)
	*p *= 0.864	*p *= 0.840
Fish-sea food		
None	1.00 (reference)	1.00 (reference)
<85 g	0.45 (0.21–0.96)	0.46 (0.21–0.98)
≥85 g	0.82 (0.43–1.59)	0.90 (0.46–1.78)
	*p *= 0.226	*p *= 0.363

^1 ^Logistic regression analysis including consumption of foods (none, below, or above median consumption) as independent variable. *P *values for linear trends as presented.

^2 ^Logistic regression analysis including consumption of foods (none, below, or above median consumption), district of residence, body mass index, energy intake, consumption of tobacco, coffee, and alcohol as independent variables. *P *values for linear trends as presented.

## Discussion

Folate has recently emerged as a key nutrient to optimising health, and inadequate folate status has been identified as risk factor for cardiovascular disease, various types of cancers, and neurocognitive disorders [1,17]. The present study described the distribution of serum folate concentrations in a healthy adult population in Crete, Greece and examined associations with dietary intakes and lifestyle habits, namely tobacco, coffee, and alcohol consumption. The relationship between serum folate and vitamin B_12 _and total homocysteine was also examined.

The results of the study should be viewed in light of some limitations. The present data used a cross-sectional design that implies that no conclusions can be drawn on the causal effect of the determinants of serum folate levels. The dietary survey was performed using one single 24-h dietary record and may thus be subject to both systematic and random bias, one source being the lack of inclusion of weekends. The regular use of vitamin supplements was not recorded and this precluded further analyses of the serum folate status. Also, the participation rate was relatively low (<20%) and selection bias cannot be excluded. Lastly, the unique sample of Hospital/Medical School personnel is both strength (as it decreases potential for confounding) and limitation (as the sample is not representative of the general Greek adult population) of the survey.

Despite these limitations, there are several findings in this study that are worth to be discussed. In this sample of apparently healthy Cretan adults, sub-optimal folate levels were found in 6.8% of men and 2.1% of women, using serum concentrations ≤7 nmol/l as a cut-off value [16]. Depending on the criteria used, the proportion of subjects with impaired folate status in other studies ranges 0–79% [18-21]. Differences in serum folate concentrations and cut-off values may be due to analytical methods, race-ethnicity differences and genetic backgrounds [1,22]. The mean daily intake of folate by the study subjects (294 μg/d in men, 247 μg/d in women) also stands between lower figures that have been reported by Plannells *et al. *[19] for Spanish adults (205 μg/d in men, 197 μg/d in women) and higher ones that Ford *et al. *[21] and Rasmussen *et al. *[23] have described for US men (343 μg/d) and Dutch women (283 μg/d in the 20–35-year-old group), respectively. In contrast to the low prevalence of sub-optimal folate concentrations, >75% of the study participants did not meet the RDI for folate (400 μg/day). Such a discrepancy between serum folate levels and dietary folate intake has been reported in several other surveys, including those where dietary folate intake was assessed by 3-day dietary records or food-frequency questionnaires, and is explained by insufficient capturing of important sources of dietary folate by dietary records [20,23-25]. As already discussed, regular use of vitamin supplements was not recorded in our survey, which may have resulted in underestimation of dietary folate intake. Finally, some researchers argue that total homocysteine is a more sensitive indicator of folate status than is serum (or erythrocyte) concentration of the vitamin [20,26]. Similarly to the results of other studies [19,20], men had higher mean intakes of dietary folate than women, but when expressed by energy intake, women had higher intake of folate than men. This might – at least in part – explain the higher serum folate concentrations in women, although other factors (e.g. hormonal, bioavailabilty of dietary folate etc.) may be responsible for this discrepancy.

In both men and women, subject age was positively related to serum folate levels. One possible explanation for this association is the higher intake of folate and folate-rich foods (especially fruits and vegetables) with increasing age among adults (data not shown). A similar trend has been reported in other studies and is generally attributed to the preference for more "healthy" or "traditional" diets among older adults [19,20,27]. With regard to the adult population in Crete, recent data indicate the gradual abandonment of the traditional Cretan diet in favour of more "westernised" diets, with consumption of higher amounts of saturated fat, meat, and cheese, and lower amounts of fruits, vegetables, legumes, and fibre [9-11].

In agreement with several other studies [28-32], serum folate was inversely related to total homocysteine concentrations. Hyperhomocysteinaemia is a well-established risk factor for atherothrombotic disease, Alzheimer's disease and other neuropsychiatric disorders, osteoporosis and hip fractures [33,34]. This overlap of medical disorders that are linked both to increased homocysteine and inadequate folate status has led to the suggestion that the disease-promoting effects of low folate are mediated through increased homocysteine levels [1,4,7,32,35]. Yet, recent data indicate that folate may act independently of homocysteine, especially through its direct effects on vascular endothelium and cellular redox status [1].

Another finding was the inverse relationship between tobacco consumption and serum folate concentrations. Smokers had significantly lower serum folate levels than non-smokers, and serum folate concentrations decreased by increasing consumption of cigarettes. A negative association between smoking and serum folate has been reported in other studies [19,28,29,32,36], and is generally attributed to the different nutritional status of smokers. More specifically, smokers tend to consume lower amounts of several vitamins, fruits and vegetables, resulting in decreased intake of dietary folate [12,37-39]. Similarly, in our study, the association between smoking and serum folate abolished its statistical significance after controlling for intakes of dietary folate, vitamins and C. It should be noted, however, that smoking may have more direct anti-folate effects, producing local vitamin deficiency in individual tissues as demonstrated by Piyathilake *et al. *[40].

In the present study, serum folate concentrations were inversely related to coffee consumption. Such a relationship has only been shown indirectly, in studies examining serum total homocysteine levels in association with coffee intake. In some of these studies, the positive association between coffee consumption and homocysteine was lost after accounting for serum folate and/or dietary folate intake, indicating a negative association between coffee intake and folate status [29-32]. As in the case of tobacco consumption, the association between serum folate and coffee was confounded by differences in nutrient intakes.

The results from the dietary survey demonstrated significant associations between serum folate and intakes of several important nutrients, including MUFA, fibre, calcium, magnesium, folate, and vitamins A, E, C, B_1_, and B_6_, independently of other confounders. A relationship between dietary folate intake and serum folate concentrations has been reported in some [18,19,23] but not all [20] studies. The favourable associations between serum folate and dietary micronutrients were reflected by differences in consumption of various foods. Individuals with higher intakes of potatoes, legumes, fruits and/or vegetables – all these foods considered major sources of folate [19,20] – had significantly decreased risk for low serum folate (below the 1^st ^quartile), compared to those with no consumption. Conversely, higher intakes of cereals and meat products were related to decreased serum folate concentrations. These findings are in accordance with those reported by both cross-sectional [41-45] and diet-intervention studies [46,47], which suggest a positive association between folate status and a dietary pattern characterized by high consumption of fruits, vegetables, legumes and low consumption of refined cereals and meat. It should be noted however, that the lower intakes of cereals and meat among the study subjects with lower serum folate levels might be simply due to substitution by other foods, namely fruits, vegetables, and legumes. Finally, an alternative explanation for the associations between serum folate levels and nutrients and foods, might be that serum folate serves as an overall nutritional biomarker and indicator of diet and health quality [48,49].

## Conclusion

Conclusively, in this cross-sectional study of healthy Cretan adults we found various demographic, lifestyle and dietary factors associated with serum folate concentrations. Considering the importance of folate in health maintenance and prevention of chronic disease, it is important to increase the public's awareness on modifiable lifestyle habits and especially diet, associated with improved folate status. In view of the high percentage of adults with inadequate intake of dietary folate, our results also emphasize the need to undertake large-scale epidemiological studies within the general Greek adult population, in order to assess the prevalence of impaired folate status, and further examine associations between dietary patterns, serum folate, and chronic disease risk.

## Competing interests

The author(s) declare that they have no competing interests.

## Authors' contributions

CH performed blood sampling, collected data, and reviewed the manuscript. GB collected data and drafted the manuscript. ML collected data and carried out the statistical analysis. In JS' laboratory biochemical measurement of serum folate, vitamin B_12 _and homocysteine was performed. JS also reviewed the manuscript. AK conceived of the study, participated in its design, and helped to draft the manuscript. All authors read and approved the final manuscript.
